# High-Resolution Seismocardiogram Acquisition and Analysis System

**DOI:** 10.3390/s18103441

**Published:** 2018-10-13

**Authors:** Fábio Leitão, Eurico Moreira, Filipe Alves, Mário Lourenço, Olga Azevedo, João Gaspar, Luis A. Rocha

**Affiliations:** 1 Center of Micro Electro Mechanical Systems, University of Minho, 4800-058 Guimarães, Portugal; id5974@alunos.uminho.pt (E.M.); lrocha@dei.uminho.pt (L.A.R.); 2 International Iberian Nanotechnology Laboratory, 4800-058 Braga, Portugal; filipe.alves@inl.int (F.A.); joao.gaspar@inl.int (J.G.); 3 Cardiology Department, Hospital Senhora da Oliveira, 4835-044 Guimarães, Portugal; lourenco.mariorui@gmail.com (M.L.); olgazevedo@yahoo.com.br (O.A.); 4 Life and Health Sciences Research Institute (ICVS), School of Medicine, University of Minho, 4710-057 Braga, Portugal; 5 3ICVS/3Bs PT Government Associate Laboratory, 4710-057 Braga, Portugal

**Keywords:** accelerometer, ballistocardiogram, MEMS, pull-in time, seismocardiogram

## Abstract

Several devices and measurement approaches have recently been developed to perform ballistocardiogram (BCG) and seismocardiogram (SCG) measurements. The development of a wireless acquisition system (hardware and software), incorporating a novel high-resolution micro-electro-mechanical system (MEMS) accelerometer for SCG and BCG signals acquisition and data treatment is presented in this paper. A small accelerometer, with a sensitivity of up to 0.164 µs/µg and a noise density below 6.5 µg/$\sqrt{\mathrm{Hz}}$ is presented and used in a wireless acquisition system for BCG and SCG measurement applications. The wireless acquisition system also incorporates electrocardiogram (ECG) signals acquisition, and the developed software enables the real-time acquisition and visualization of SCG and ECG signals (sensor positioned on chest). It then calculates metrics related to cardiac performance as well as the correlation of data from previously performed sessions with echocardiogram (ECHO) parameters. A preliminarily clinical study of over 22 subjects (including healthy subjects and cardiovascular patients) was performed to test the capability of the developed system. Data correlation between this measurement system and echocardiogram exams is also performed. The high resolution of the MEMS accelerometer used provides a better signal for SCG wave recognition, enabling a more consistent study of the diagnostic capability of this technique in clinical analysis.

## 1. Introduction

Each year, cardiovascular diseases (CVD) cause 3.9 million deaths in Europe, which account for 45% of all deaths. In 2015, more than 85 million people in Europe were living with CVD and, over the past 25 years, the absolute number of CVD cases has increased [1]. Consequently, cardiac monitoring is important to evaluate cardiac system performance and to prevent or detect cardiovascular diseases.

Ballistocardiogram (BCG) and seismocardiogram (SCG) are non-invasive measurements of the recoil forces of the body in response to the ejection of blood from the heart and the movement of blood through the vasculature [2]. The term seismocardiogram is generally used for local vibrations recordings when the accelerometer sensor is placed close to the heart at the apex or on the sternum. In contrast, a ballistocardiogram shows recordings of the overall body movement when the sensor is placed close to the subject. This is mostly placed on items which support the body, such as beds, chairs or weighing scales. 

In the last few decades, technological advances have enabled the development of sensors such as micro-electro-mechanical system (MEMS) accelerometer sensors. These sensors have been used to create BCG and SCG measurement systems with better diagnostic capacity. The BCG technique, which fell into disuse following the development of the electrocardiogram (ECG), echocardiogram (ECHO) and magnetic resonance, seems to have re-emerged with new perspectives for its clinical use [2]. 

Recent hardware developments for SCG and BCG acquisition systems and the improved measurement methodologies reflect this reappearance process. The main advantages of BCG and SCG systems, such as a wearable SCG based on MEMS accelerometers [3] or an ear-worn BCG device [4], are its portable feature for continuous daily monitoring. Other approaches based on weighing scales [5,6,7], which are domestic items that take advantage of the user’s longitudinal position, have also been presented. However, these impose some practical limitations with regards to long measurements and noise floor exposition. Chair-based systems have also been reported [8,9]. These systems are more comfortable and reduce the involuntary movements of the subject during measurement. Moreover, wheelchair-based systems allow a daily cardiac monitoring of reduced mobility patients. Nevertheless, this technique shows a reduction in signal amplitude when compared to the other measurement systems previously introduced. A study reported in Reference [10] explored BCG measurements in a car seat. The results were not conclusive since the BCG signal became undetectable whilst driving, due to the external acceleration distortions measured by the sensor. The placement of sensors onto beds which has also been explored, enables the evaluation of sleep stages and the detection of diseases in a non-clinical environment [11,12]. This procedure does not require direct contact with the patient, which reduces involuntary disturbances during sleep and is therefore more advantageous than the electrocardiogram technique [2].

Echocardiogram and magnetic resonance exams are useful for sporadic diagnosis, assessment and monitoring. However, they are expensive exams requiring an equipped room in a hospital or clinical center and experienced operators to perform the exams.

Some studies have demonstrated that through BCG and SCG signals it is possible to estimate the respiratory frequency as well as the heart rate, showing correlations with other monitoring and clinically validated systems. Regarding BCG signals, it is possible to find in the literature correlations between the amplitude of BCG signals’ waves and the left ventricular function allowing estimation of the stroke volume and the cardiac output [13]. In addition, when BCG is simultaneously measured with ECG, the delay between these signal waves shows correlations which enable the estimation of parameters such as the pre-ejection period (PEP) or pulse transit time (PTT) [2,14]. However, SCG and BCG signals reveal an inter-subject vulnerably regarding body mass index, gender, and health conditions. For instance, patient movements or muscle related diseases can lead to a misunderstanding of SCG signal features [2,15].

The use of these signals to investigate heart rate variation (HRV) and blood pressure (BP) is also addressed in Reference [2]. HRV measurements make it possible to evaluate sleep efficiency and the autonomic nervous system. Since a plausible assessment of these parameters requires long data acquisition periods, an examination based on BCG, due to the non-contact characteristics, gains relevance and can be an efficient substitute for ECG. 

Recent literature shows growing confidence in the applicability of SCG over BCG in clinical practices [16,17,18,19]. This has led to the development of new methods and algorithms to perform SCG waves recognition and peaks detection and their association with cardiac events. In References [18] and [20] SCG fiducial points are detected: The isovolumetric moment (IM), the aortic valve opening (AO), and the aortic valve closure (AC) which, when combined with ECG signals, can be used to estimate cardiac events such as systolic and diastolic time, PEP and left ventricle ejection time (LVET). In Reference [17] a multi-channel SCG spectrum measurement system was proposed and new waves associated with cardiac events were detected. 

Despite recent advances, and according to the review carried out in Reference [2], BCG and SCG signals still do not denote conciseness in terms of their biological significance or clinical relevance, and the correlations made by most recent studies are conducted mainly from healthy subjects. In addition, reference values determining a healthy subject and correlating the distinction in BCG and SCG signals according to the body size or age are still unknown, as well as the relation between the results obtained from different measurement methodologies. It is also difficult to establish a specific correlation between different heart diseases and which signal disorders are induced into ballistocardiogram and seismocardiogram waves [13]. Put simply, there is still room for improvements to be made on both hardware types to acquire BCG and SCG signals (better signal-to-noise ratio acquisition systems) and the corresponding clinical interpretations of the acquired data.

Typically, in these studies, commercial MEMS accelerometers are used for the SCG signal detection. These accelerometers present a measurement range of around ±1 g or ±2 g and a bandwidth of up to 1 kHz. The acquisition system resolution is limited by the noise performance of these accelerometers, with noise densities above 98 µg/$\sqrt{\mathrm{Hz}}.$

Advances in microtechnology and the development of innovative MEMS designs have enabled the creation of high-resolution accelerometers that achieve micro-g resolutions. A time-based MEMS accelerometer presented in Reference [21] uses time measurement as the acceleration transduction mechanism, and as demonstrated, enables the measurement of micro-g signals with high-resolution (<3 µg/$\sqrt{\mathrm{Hz}}$). The main advantage of this approach is that time measurements can be very accurate compared to capacitive readout circuits used in conventional capacitive accelerometers. On the other hand, sensor resolution can be improved with better time measurement, and sensor bandwidth can be extended by decreasing pull-in time transition [21]. 

Most BCG and SCG signals’ energy are located in bands below 30 Hz and have amplitudes of around 10 mgp-p [2,22]. For the typical commercial MEMS accelerometers used in the reported literature, this implies (for a bandwidth of 30 Hz and a noise density of 98 µg/$\sqrt{\mathrm{Hz}}$) a resolution of the acquisition system around 0.54 mg. This gives roughly a 5-bit resolution for the full acquisition system which strongly compromises the signal analysis. These conditions increase the opportunity of implementing an acquisition system for SCG (and BCG) signals that incorporates a MEMS accelerometer based on pull-in time measurement, extending its application to a more consistent study of the diagnostic capability of this technique.

In this work, an integrated and improved version of the accelerometer presented in Reference [21] is used to evaluate its performance on a wireless SCG and BCG acquisition system. The full SCG analysis system that also includes an ECG synchronized signal acquisition, calculates several SCG parameters (based on the measured signals) that are compared to parameters and indexes calculated through an echocardiogram system performed at similar conditions as ECG and SCG acquisition. This procedure leads to an improvement (due to the much better signal-to-noise ratio and system resolution) on the SCG peaks recognition and a more consistent study of SCG technique and its diagnostic capability.

## 2. System Description

### 2.1. SCG and ECG Acquisition Device

**Figure 1:**
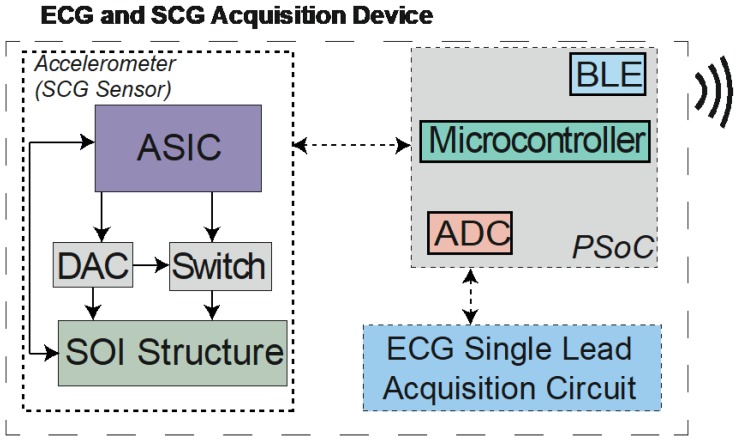
The block diagram of the acquisition device.

**Figure 2:**
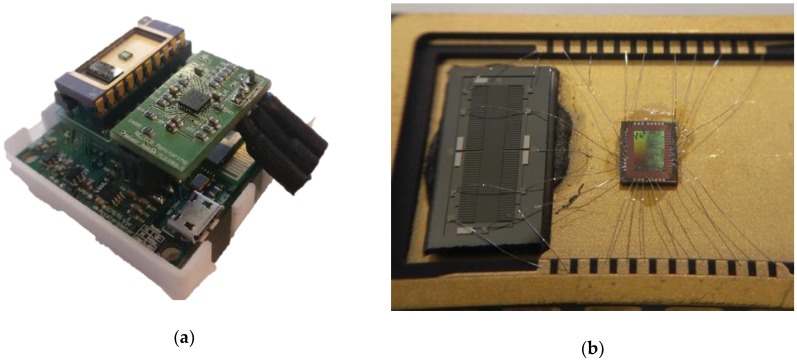
The acquisition device: (**a**) The system main board which contains the ECG acquisition subsystem and SCG sensor and (**b**) the integrated acceleration sensor (MEMS and ASIC).

The SCG and ECG acquisition system introduced in this work is a small system (40 × 40 × 20 mm^3^) with wireless capabilities, and can be applied in different measurement contexts. Figure 1 shows the measurement system block diagram and its main components. The core component is the time-based high-resolution accelerometer (Figure 2b) that includes a differential actuation mechanism (as opposed to a single-ended approach used in Reference [21]) and an application-specific integrated circuit (ASIC) for accelerometer operation control. Three main subsystems can be identified: The acceleration sensor for the SCG signals measurement, which is composed of the silicon on insulator (SOI) microstructure, controlled by an ASIC. The ASIC controls the MEMS element through a digital-to-analog converter (DAC) and a switch for the structure actuation; the ECG signal acquisition circuit is connected to an analog-to-digital converter (ADC) that allows the reading of a single lead ECG; and the Programmable System on Chip (PSoC) that performs the reading of the signals data, synchronization and transmission over the integrated Bluetooth Low Energy (BLE) peripheral.

Figure 2a presents the full acquisition system and Figure 2b shows a detailed image of the Silicon-on-Insulator MEMS and ASIC on a dual in line ceramic package (it should be noted that the DAC and switches are outside the ceramic package).

The SOI structure has parallel-plates for sensing and for actuation which are symmetrically placed in each direction, and it has very similar characteristics to the devices used in Reference [21]. The ASIC (implementation details in Reference [23]), is connected to the microstructure, and is responsible for the reading of inertial mass’ position and pull-in phenomenon detection and control of the external actuation system (DAC and switch). The ASIC also performs the pull-in time counting and triggers the interrupt signals to the microcontroller when time data are ready.

An ECG acquisition system is also integrated for data correlation and to provide the possibility to calculate more parameters. The ECG signal acquisition circuit is based on the AD8232 (from Analog Devices, Norwood, MA, USA) consisting of a fully integrated single-lead ECG front-end module. The microcontroller (a PSoC from Cypress Semiconductor Corporation, San José, CA, USA) receives the ASIC’s pull-in time data (through an I2C line) and the ECG signal (internal ADC readings) and sends it to a PC running the analysis software through BLE. The system can be powered from any 5 V source, such as a small battery.

### 2.2. The Analysis System

**Figure 3:**
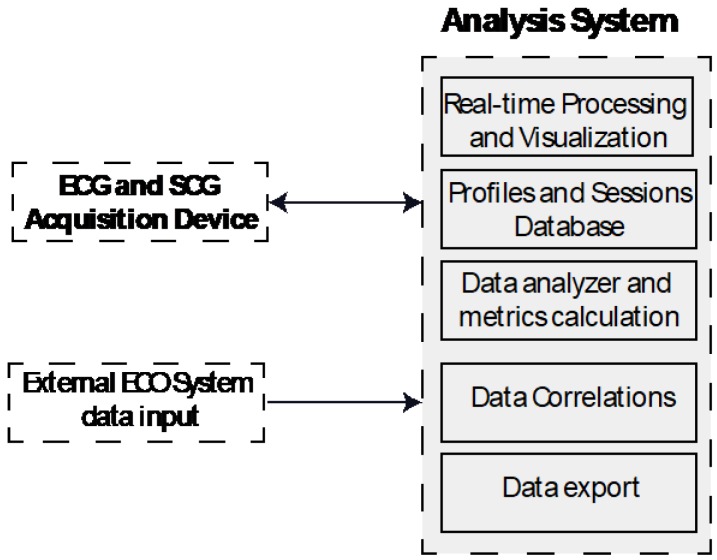
Analysis system block diagram.

The real-time acquisition and analysis software for measured data evaluation has been developed using MATLAB. A simplified block diagram of the full acquisition system (hardware and software) is shown in Figure 3.

Among other features, the software allows the creation of user profiles with personal information (gender, age, weight, height, among others) that can be correlated. It is also possible to create classifier groups (tagged by pathologies, for instance) to which each profile can be associated with. Consequently, exams performed using the acquisition system are linked to each specific user’s profile.

The acquisition device data is received over the BLE USB dongle connected to the computer and during each session, the synchronously acquired SCG and ECG signals are processed for real-time visualization. Simultaneously, the measured data are stored in their raw conditions, preventing information loss. This also allows the improvement of existing algorithms, or the creation of new algorithms for the session analysis. 

**Figure 4:**
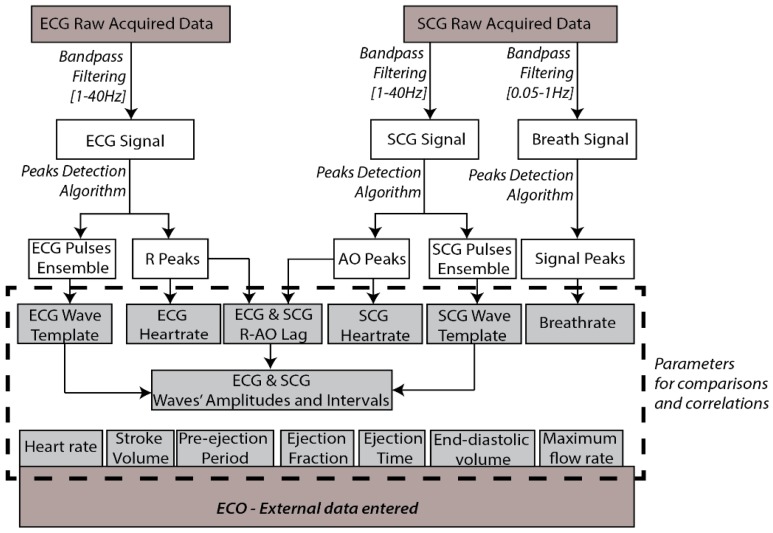
Data processing and procedures for ECG and SCG parameters calculations.

Once the session is completed, the data are available to be processed. Figure 4 shows a block diagram which summarizes the procedures for signals’ parameters calculations over the acquired ECG and SCG raw signals.

A zero-phase digital bandpass filter is applied to the ECG raw data, providing the ECG singular waves recognition. A peak detection algorithm is used to find the signal’s maximums, matching the ECG R-waves and estimating the heart rate value. The singular ECG waves are overlapped, and an ECG template wave is calculated based on the ensemble mean wave.

In turn, the SCG signal is filtered to dual specific bandwidth frequencies, (0.05–1 Hz) and (1–40 Hz). This allows signals related to breathing, and mechanical SCG waves to be distinguished. The maximums of both filtered signals are calculated using an algorithm based on peak detection, and are used to estimate breath rate (BR) and heart rate (HR) values. Similar to the ECG analysis, the SCG ensemble is computed and a template wave is calculated based on the ensemble’s average. 

Considering the opportunity of estimating cardiac events through a synchronized measurement of ECG and SCG signals, (evidenced in the introduction) an analysis which combines these signals can also be performed. The ECG and SCG waves, interpolated to the equivalent sampling frequency, show a time lag that can be identified through the delay between R and AO peaks, due to their higher amplitude. Then, the previously estimated delay, applied to the ECG and SCG template waves, is used to estimate all the remaining signal peaks’ lags. This can be seen in Figure 11 which demonstrates part of the results of a preliminary study that is presented later.

To improve the SCG diagnostic capability, metrics extracted by an echocardiogram system can be inserted in the software and used to perform correlations. In case statistical analysis is required on a different platform (on an SPSS platform, for instance), all database content, such as user and session information, can be fully exported.

## 3. SCG Acquisition System Characterization

**Figure 5:**
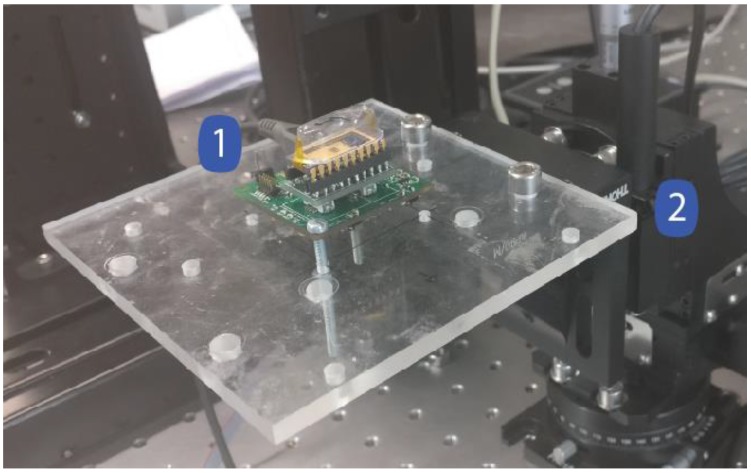
Experimental setup for the acquisition system characterization.

The performance evaluation of the acquisition system is directly linked to the accelerometer performance, and therefore the acquisition system characterization includes the MEMS sensor evaluation (using the experimental setup depicted in Figure 5).

To perform measurements using constant input accelerations, the acquisition system board (1) was horizontally attached to a high precision motor (CR1-Z7, THORLABS) and the sensor direction positioned in a perpendicular angle relative to the gravitational force. Thus, the motor angle (2) variation induces changes in the input external acceleration.

### 3.1. Sensitivity Characterization

Sensitivity and bandwidth in a time-based MEMS accelerometer depend both on the device characteristics and the actuation voltage applied on the actuators’ parallel-plates. The accelerometer sensitivity and bandwidth for different actuation values were measured for a range between ±50 mg (enough for SCG measurements), and the sensitivity results are summarized in Table 1.

**Figure 6:**
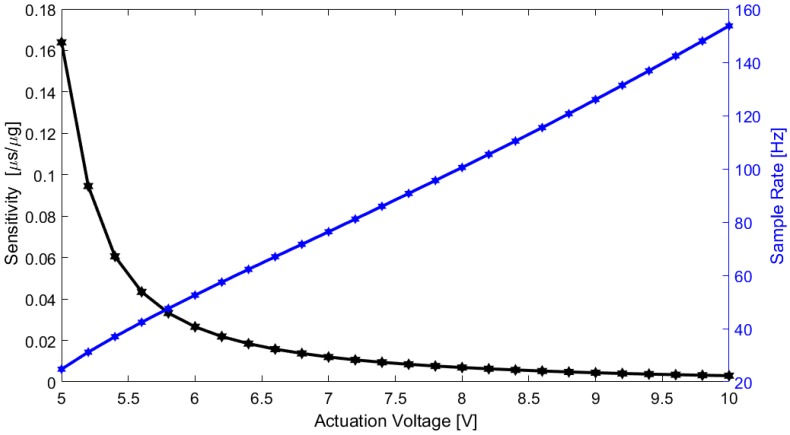
Sensor sensitivity and bandwidth characterization.

A graphical representation of the accelerometer sensitivity variation and bandwidth is presented in Figure 6. As expected, both the sensitivity and bandwidth depend on the actuation voltage (it is possible to tune the accelerometer considering the targeted application), and a trade-off is required between the sensitivity and bandwidth. 

### 3.2. Noise Level Measurement

**Figure 7:**
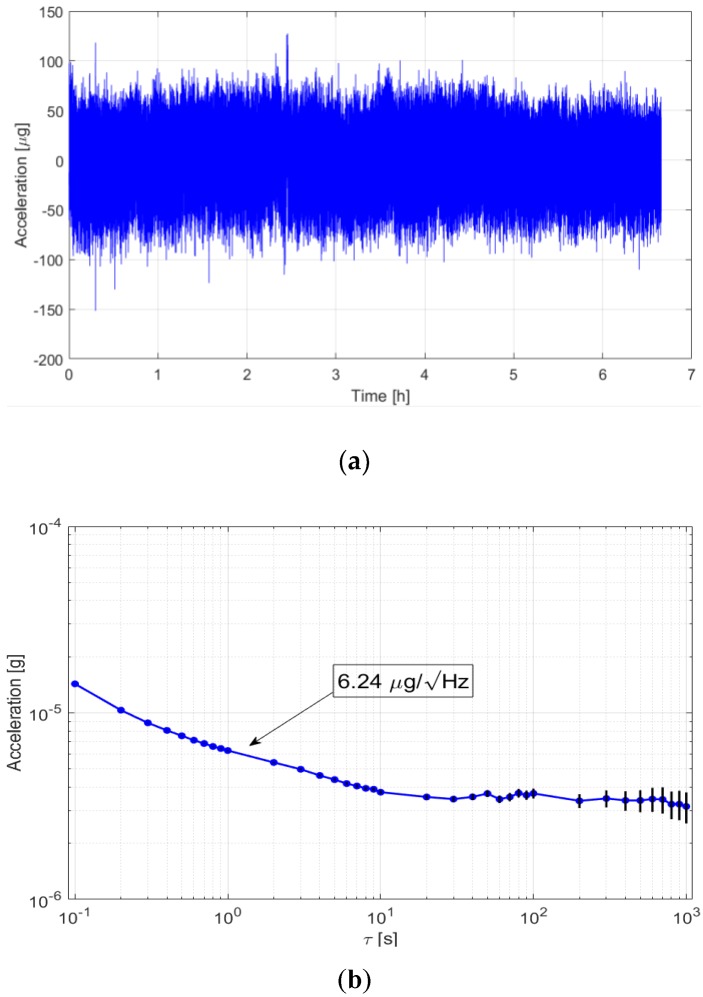
Measurement results of the (**a**) long-term test and (**b**) Allan deviation analysis.

One of the critical characteristics, also due to the very small amplitude of the SCG signals, is the accelerometer noise density. A noise characterization was done by performing a 6 h acquisition test (in the absence of input acceleration) for an actuation voltage of 5.00 V. The long-term measurements are shown in Figure 7a, and the related Allan variance analysis is shown in Figure 7b. The Allan variance analysis shows a noise floor below 6.5 µg/$\sqrt{\mathrm{Hz}}$ and a bias instability of 3.54 µs for a 20-s integration time. 

It should be noted that the noise is mainly due to mechanical thermomechanical noise and therefore, unlike the sensitivity and bandwidth, does not depend on the actuation voltage. The use of this accelerometer enables an SCG acquisition system with a 9-bit resolution (for a bandwidth of 30 Hz and 10 mgp-p signal).

## 4. SCG Acquisition System Performance over a Preliminary Clinical Study

To effectively test the developed wireless acquisition system with real SCG signals and validation of the analysis software for ECG and SCG waves recognition, a preliminary clinical study was performed. The study and measurements took place at the Cardiology Department in Hospital Senhora da Oliveira in Guimarães, Portugal.

### 4.1. Subjects Selection

Twenty-two participants (5 healthy subjects and 17 subjects with diagnosed cardiovascular diseases) served as voluntary subjects to the study covering five groups. One of the groups are healthy subjects and the remaining ones were divided by the following pathologies: Dilated cardiomyopathy (DCM), myocardial infarction (MI), aortic stenosis (AS) and hypertrophic cardiomyopathy (HCM). At the beginning of the exam, each participant consented to the exam procedures through an approved informed consent form and their personal information was kept in anonymity. Table 2 summarizes the subject characteristics.

At this preliminary study stage, finding subjects with a singular and well-defined cardiac pathology (within the pathologies mentioned) was the principal motivation for the selection conditions. 

### 4.2. Measurement Methodology

**Figure 8:**
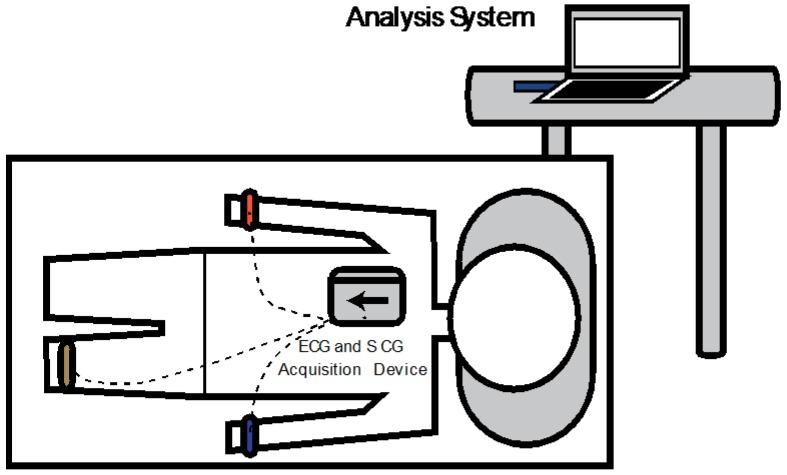
Measurement methodology and device position.

A bed-based measurement methodology which comprised a subject in the supine position was chosen. This position enabled the measurements to be performed in a stable position, even for the most debilitated subjects. It also created similar measurement conditions during the measurement process for the different subjects. Figure 8 shows the sensor’s location and the subject’s position during the measurements. 

For SCG signal detection the developed system was placed near to the heart on the sternum. The ECG signal was measured through electrodes placed on arms and legs linked to the system main board. The SCG and ECG measurements were performed during 120 s for each subject using an actuation voltage of 10 V (2.98 ms/g sensitivity and 155 Hz bandwidth) for the MEMS accelerometer and using a sampling rate of 248Hz for the ECG sensor. Under a similar position, an echocardiogram exam was also performed for each subject.

### 4.3. Session Analysis

After a measurement session was completed, the data were ready to be analyzed and the SCG and ECG data parameters could be estimated. The results for each SCG and ECG measurement session are presented in an individual panel within the analysis software, and a joint ECG and SCG panel is also available for signal comparison. 

#### 4.3.1. ECG Analysis

**Figure 9:**
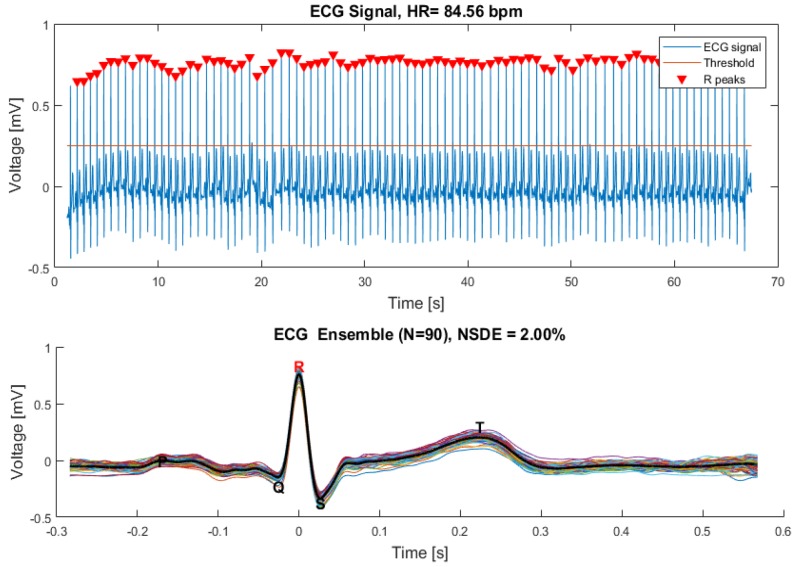
ECG analysis panel.

The ECG signal analysis was performed through the ECG analysis panel depicted in Figure 9. The R waves are visible, and the HR value based on those waves can be estimated by a peak detection algorithm. The ECG waves ensemble was used to determine the ECG template wave and the remaining PQST waves. A metric called Normalized Standard Deviation from the Ensemble (NSDE) [22] was used to calculate the standard deviation between individual waves and the ensemble mean wave, thus allowing the quantification of the ECG repeatability. These parameters were automatically calculated from a selected time interval (enabling the skipping of inadvertent artifacts captured during the session).

#### 4.3.2. SCG Analysis

**Figure 10:**
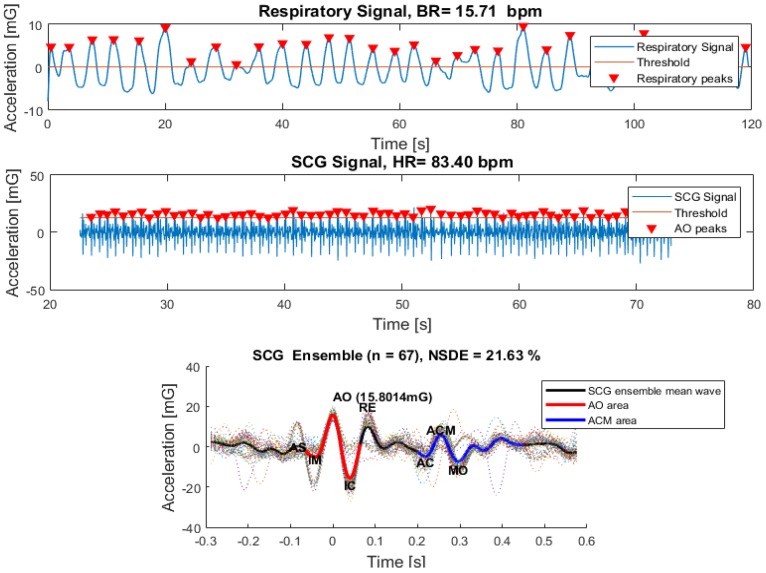
SCG analysis panel.

On another panel, an individual SCG signal analysis was also performed. The results of the SCG analysis panel are shown in Figure 10. The raw data filtered over two bandwidths allow the distinction between breathing and mechanical SCG signals. 

An algorithm based on the detection of peaks applied to breathing signals, runs the estimation of the respiratory rate value. The detection of SCG singular waves was more complex as compared to ECG. That distinction was done based on the calculation of the SCG signal envelope. Then, the maximum peak of each singular wave was defined as the first maximum inside the envelope area. These peaks’ detection algorithm allows the estimation of the heart rate value and the performance of the SCG waves disassembly. Similar to the ECG analysis, an SCG overlapped ensemble was also calculated and the SCG template wave was extracted based on the ensemble mean. The SCG pulse deviation variation was estimated through the calculation of the NSDE. 

The SCG wave analysis carried out in References [18,20], demonstrated the existence of two SCG signal periods: The cardiac systolic (AO area) and diastolic (ACM Area) periods. To capture those regions of interest, the systolic period and the maximum peak (AO) were defined from the maximum peaks of the SCG template. The diastolic event, which occurs in the interval between 200 and 400 milliseconds from the AO was determined through the identification of the highest peak (AC) and the lowest valley (MO) in the defined interval. After AC and MO identification, the diastolic event was clearly defined.

#### 4.3.3. ECG and SCG Analysis

**Figure 11:**
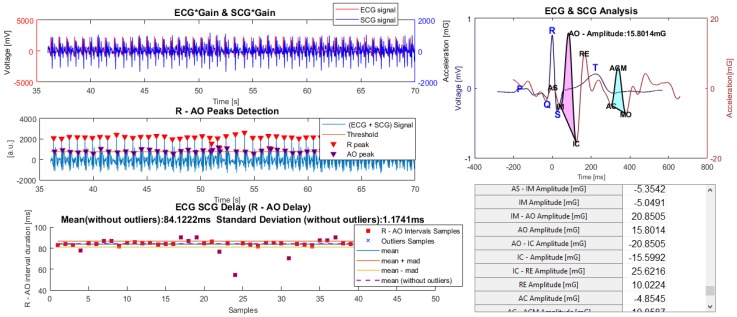
ECG and SCG analysis panel.

The analysis of the combined ECG and SCG signals was performed on a third panel (Figure 11). Here, the ECG and SCG signals were interpolated to the equivalent sampling frequency and the time lag between their waves was calculated through the R-AO peaks’ delay. It should be noted that the AO wave appears closely after the R peak occurrence. Therefore, the intervals were detected through the individual signals’ amplification followed by their sum. Finally, the maximum peaks and their nearest, matching the R-AO intervals samples, were identified. Measuring R-AO intervals variability based on mean and median absolute deviation lead to the estimation of a reference R-AO. The ECG and SCG template waves distanced by this R-AO reference interval, allows the estimation of interval delays between all the previously demarked peaks. A set of parameters related to the echocardiogram exam such as the heart rate, stroke volume, pre-ejection period, ejection fraction, left ventricular ejection time, end-diastolic volume and maximum flow rate can be inserted and attached to the respective session and used for correlations. All the analysis’ variables (calculated thresholds and selected intervals), and the extracted data from each individual panel were saved for each session.

#### 4.3.4. Data Comparison and Correlations from Measurement Systems

The measured parameters from the sessions, i.e., the data obtained from the developed system (ECG and SCG) and the inserted parameters from ECHO, are available for graphical visualization and comparison. Some correlations based on the results obtained from the 22 subjects’ sessions, that use the SCG signal parameters, are presented in this section. The strength and direction of the linear relation uses the Pearson’s linear correlation (α = 0.05).

**Figure 12:**
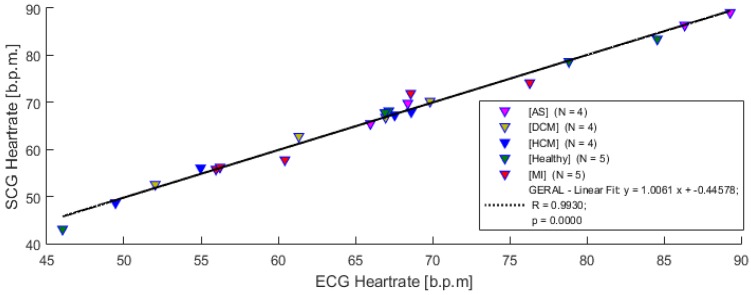
Heartrate correlation from ECG and SCG systems.

Figure 12 compares the extracted heart rate from the measured ECG and SCG signals (acquired with the developed acquisition system). 

This result shows a high correlation coefficient, *R* = 0.993 (*p* < 0.05), clearly evidencing that SCG signals can be used to monitor heart rate from the several measured subjects.

**Figure 13:**
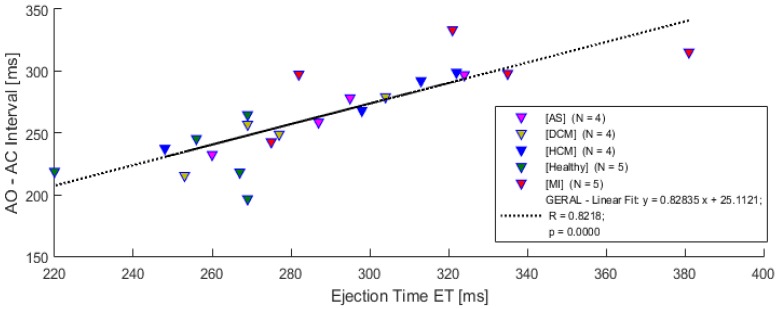
Left ventricular ejection time vs. AO-AC interval from SCG and ECHO.

A comparison between the AO-AC interval measured from SCG waves and left ventricular ejection time extracted from the ECHO exam is depicted in Figure 13. In this preliminary study, a strong association with the ejection time measured from ECHO and the AO-AC interval extracted from SCG signals, is evidenced by the correlation coefficient (*R* = 0.8218 (*p* < 0.05)). This result reveals that the AO-AC interval might exhibit a linear dependence in all subjects, including the ones with diagnosed pathologies.

**Figure 14:**
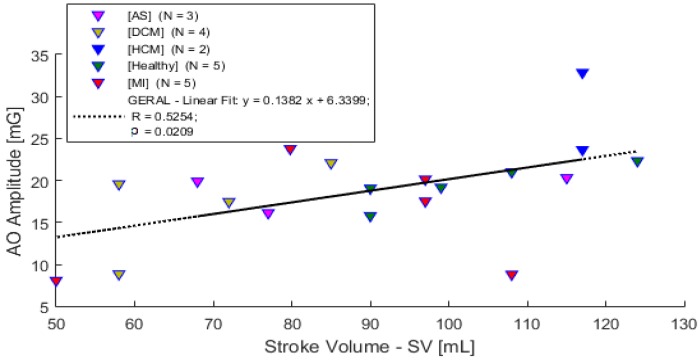
Stroke volume vs. AO wave amplitude from ECHO and SCG.

A comparison between the AO peak amplitude and the stroke volume from ECHO is presented in Figure 14. When the stroke volume extracted from the ECHO exam is compared to the AO wave amplitude from the SCG signal, a moderated correlation is observable (*R* = 0.5254 (*p* < 0.05)). 

As described in the literature, the SCG signal might have a high dependence on subject characteristics or muscle related diseases. In this trial study, all the subjects, even the ones with cardiac diseases, were included in the analysis. Table 2 shows that there is a strong variation in the subjects’ physiological characteristics and consequently, future work with more subjects is required for a better understanding of this and other aspects.

## 5. Conclusions and Future Work

A high-resolution wireless acquisition system for SCG and ECG signals was presented in this paper. The SCG signal was measured using a pull-in-time based accelerometer with a sensitivity of up to 0.164 µs/µg and a noise density below 6.5 µg/$\sqrt{\mathrm{Hz}}$. These characteristics make this system suitable for SCG, or even BCG, measurement applications, with a good SNR and resolutions of up to 9 bits (a huge improvement when compared to previous systems used in the reported literature). 

Software (analysis system) for data acquisition, visualization and processing was also developed. The software includes several capabilities such as user profile creation, group classification and real-time data visualization. Data extracted from an echocardiogram external exam can be also inserted for data correlation, and comparisons over the extracted parameters from the several measurement systems (ECG, SCG and ECHO) can be performed.

A preliminary clinical study of 22 subjects (some healthy and some with CVDs) in a clinical environment was performed to test the capability of the developed acquisition system (hardware and software). Data collected from an ECHO exam performed on the same subjects, were also included in the study. Comparisons between the measurement systems for heart rate measurement, left ventricular ejection time and stroke volume revealed promising results, causing the use of this system to be a more consistent study of the SCG technique.

Such a high-resolution acquisition system has several advantages. The system enhances the acquired data which might lead to the detection of new peaks or features that are related to cardiac events (features that, on the current systems, are masked by noise), bringing about added information that can be clinically relevant. 

Future work will include increasing the number of subjects in the study to construct a more robust database for data analysis and comparisons. A larger number of healthy subjects allow a better estimation of the reference values for the cardiac events’ intervals and the definition of typical healthy SCG waves. Once these are defined, one can see if any of the reference values for the healthy subjects are changing for groups with pathologies. The correlation of the SCG signal with a biomarker echocardiogram sample frames, which are both synchronized by the ECG signal, can also be added to the analysis system, thereby improving the overall SCG analysis.

## Figures and Tables

**Table 1:** Accelerometer sensitivity characterization.

Actuation Voltage (V)	Sensitivity @ ±50 mg (µs/µg)
5.00	0.16392
5.40	0.06059
5.80	0.03326
6.00	0.02659
6.20	0.02190
6.40	0.01845
6.60	0.01581
6.80	0.01374
7.00	0.01200
8.00	0.00693
9.00	0.00440
10.00	0.00298

**Table 2:** Subjects characteristics (µ—mean, 𝜎—standard deviation).

Classifier	No.	Gender		Age		Weight (kg)		Height (cm)		BSA (m²)	
		M	F	µ	𝜎	µ	𝜎	µ	𝜎	µ	𝜎
Healthy	5	5	0	27.2	3.6	83.20	9.52	180.8	7.29	2.04	0.16
AS	4	3	1	64.0	15.1	70.50	11.03	163.8	7.46	1.76	0.12
DCM	4	1	3	62.0	17.05	68.75	10.40	157.3	8.85	1.70	0.17
HCM	4	3	1	62.3	13.05	79.75	20.55	168.5	9.75	1.89	0.29
MI	5	4	1	69.6	8.79	76.40	10.78	163.6	4.62	1.82	0.11
Total	22	16	6	56.2	19.58	76.09	12.78	167.3	10.74	1.85	0.20
